# Barriers to Liposomal Gene Delivery: from Application Site to the Target

**Published:** 2016

**Authors:** Mostafa Saffari, Hamid Reza Moghimi, Crispin R Dass

**Affiliations:** a *Department of Pharmaceutics and Nanotechnology, School of Pharmacy, Shahid Beheshti University of Medical Sciences, Tehran, Iran. *; b *Current Address: Department of Pharmaceutics, School of Pharmacy, Islamic Azad University, Tehran, Iran.*; c *School of Pharmacy, Faculty of Health Sciences, Curtin University, Perth, Australia*

**Keywords:** Gene therapy, Drug delivery, Liposomes, Epithelial barriers, Cellular uptake and release, Degradation, Targeting

## Abstract

Gene therapy is a therapeutic approach to deliver genetic material into cells to alter their function in entire organism. One promising form of gene delivery system (DDS) is liposomes. The success of liposome-mediated gene delivery is a multifactorial issue and well-designed liposomal systems might lead to optimized gene transfection particularly in vivo. Liposomal gene delivery systems face different barriers from their site of application to their target, which is inside the cells. These barriers include presystemic obstacles (epithelial barriers), systemic barriers in blood circulation and cellular barriers. Epithelial barriers differ depending on the route of administration. Systemic barriers include enzymatic degradation, binding and opsonisation. Both of these barriers can act as limiting hurdles that genetic material and their vector should overcome before reaching the cells. Finally liposomes should overcome cellular barriers that include cell entrance, endosomal escape and nuclear uptake. These barriers and their impact on liposomal gene delivery will be discussed in this review.

## Introduction

Gene therapy is a therapeutic modality that relies on successful delivery of nucleic acid agents to deliver genetic material into cells to alter their function in entire organism. The genetic material can be either DNA or RNA, and the altered function can be an increase or decrease in the production of a protein. The protein is not restricted to being a natural product of the host cells, and the host cells are not required to be functioning as a part of the whole (1). Use of antisense oligonucleotides, ribozymes, DNAzymes, plasmid DNA and siRNA as genetic material in treatment of diseases is because of their ability to modulate gene expression (2, 3).

 As for other compounds, the main barrier to gene therapy is achieving delivery of the genetic material in sufficient quantities to the correct target sites of action and for the desired timeframe to achieve the desired level of therapeutic effect. A main role of gene delivery research is to develop clinically relevant vectors that can be used to combat elusive diseases such as AIDS (4,5). Development of an ideal carrier for effective delivery of therapeutic agents into diseased sites has always been a prime objective in any sort of therapy (6, 7). The carriers should selectively and efﬁciently deliver a gene to target cells with minimal toxicity to otherwise healthy normal tissue. Genetic materials are high molecular weight, polar compounds that do not permeate the biological barriers easily and require special carriers and targeting method. 

Viruses are efﬁcient in transducing cells but their toxicity is still a big issue. Nonviral gene delivery systems are considered as encouraging substitutes to viral vectors. These systems are based on entrapment or electrostatic interactions of anionic genetic material and cationic carriers, which provide protection from enzymatic degradation. Such interactions proffer slightly cationic particles that facilitate binding to the anionic cell surface and promote cell uptake. It is well known that charged particles in general have increased interactions with the membrane while uncharged ones like PEGylated nanoparticles have reduced interactions by virtue of their steric hindrance (7-9).

Non-viral gene transfection carriers can be categorized into lipid-based and polymer-based systems. Polyethylenimine is an example of polymer-based systems and has emerged as a potent candidate for gene delivery to the lung (7, 8). . Liposomes, the subject of the present review, belong to the lipid-based group (10). Liposomes are widely used in gene delivery. Successful examples of liposomes include Lipofectamine 2000, Lipofectin and Lipofectace (11), have been successfully used for gene delivery in culture, in animals and in patients enrolled in phase I and II clinical trials and seem to be more efficient than naked gene delivery (12-14). All of these are cationic liposomes and unlike anionic or electroneutral liposomes, cationic liposomes target the vasculature of tumours selectively (15).

**Figure 1:**
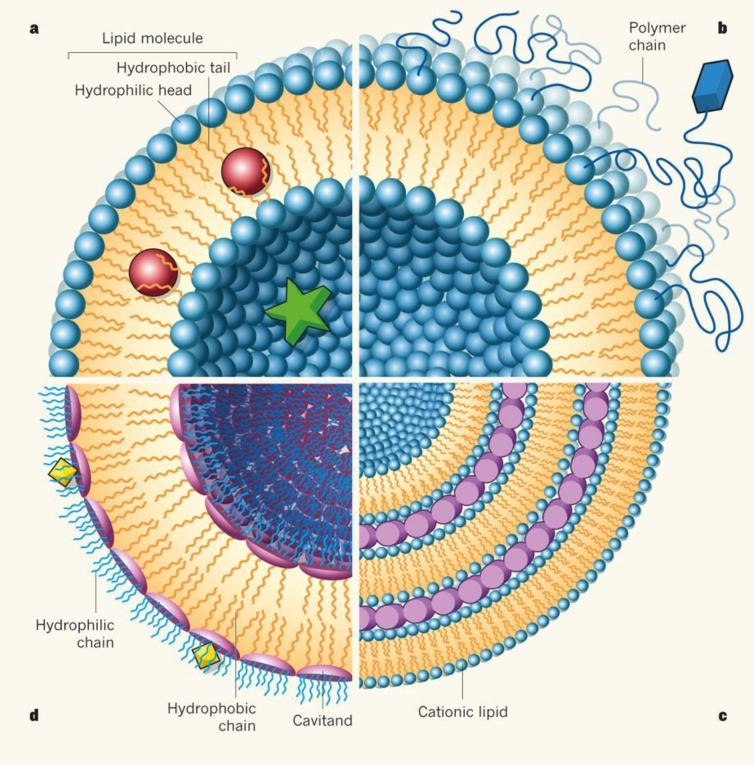
Schematic structure of liposomes showing different compositions (129

Liposomes (Figure 1) are composed of one or more simple or functional concentric lipid bilayer membranes that sandwich hydrophiliic spaces in amongst them. Solubility and method of formulation will define that drugs can be incorporated in either the aqueous or the hydrophobic phase (6, 7). Morphology variation and size difference of liposomes may vary based on lipid composition of the liposomes, formation condition of vesicles, the proportion of lipids to genetic material, intrinsic molecular weight and structure and size of the genetic payload (13, 14 and 16). Liposomes might be applied in other formulations such as gels for transdermal drug delivery or bioerodible hydrogels for controlled release of nanoparticles to increase their durability in application site, plasma or other organs (17, 18). Such formulations also affect the properties and fate of liposomes.

Genetic material works at the cellular level and, therefore, depending on the route and method of application, they face different barriers that can be categorized as pre-systemic, systemic and cellular/subcellular barriers. The pre-systemic barriers to gene delivery, which dictate such parameters as pharmacokinetics, bioavailability and targeting, depend upon the route of administration (19, 20). In systemic delivery, nuclease-mediated degradation is considered the most important barrier for nucleic acid drugs. This obstacle can be prevented by complex of such agents with a suitable component of carrier, which therefore facilitates cellular uptake. For example, Stabilized Antisense Lipid Particles or SALPs (21) and dendrosomes (22, 23) are special liposomes that are designed and investigated for this purpose and have been used for delivery of short single strand oligodeoxy nucleotides (AsODN) which target PKC-α in non-small cell lung cancer (NSCLC). Studies indicate that the encapsulating oligonucleotides in SALP and dendrosomes form stable nanoparticles in a highly reproducible manner that promote efficient cellular uptake and most importantly displayed no non-specific toxicity (21-23).

In the absence of serum, cationic particles interact with cells and lead to efficient gene delivery, although serum components may affect their function when applied systemically. Serum components interact with the particles, break their structure, confine them to blood compartment and activate complement after aggregation (24). Some of these barriers have been bypassed by administration of liposomes intra-arterially upstream to the tumour. This method looks to be dependent on the lipid composition of liposomes (25).

To act at the cellular level, genetic materials (DNA or RNA) must cross the cell membrane. Due to their inherent high molecular weight and polarity, these materials cannot naturally cross the cell membrane by passive diffusion at therapeutically effective doses (24). Therefore, the main pathway remains to be endocytosis (26, 27). In this pathway, the genetic material should be able to escape lysosomal degradation. Nuclear entry is essential for plasmid DNA while RNA can act in the cytosol and its therapeutic effect may be achieved more easily than DNA (28). 

In this review, these barriers and their impact on liposomal gene delivery would be discussed.

*Presystemic barriers*

Like other formulations, genetic materials also can be delivered through routes other than IV, either for local effects or for entering the systemic circulation after passing the epithelial barriers, which are called presystemic barriers in here. This section focuses on gene delivery through different permeation pathways considering their structure, limitation, permeation and special obstacles.

*Oral delivery*

The oral route is one of the most attractive methods of drug delivery for majority of therapeutic agents, however, oral bioavailability of genetic materials is too low to provide therapeutic effects. GIT epithelial barriers (stomach and intestine) are lipophilic membranes and provide a strong barrier against absorption of genetic materials, which are large and charged molecules (29). Gastric acidity is another problem as genetic materials are not stable at very low pH values. Presence of large number of multidrug resistant proteins (such as p-glycoprotein, p-gp) and the multispecific organic anion transporter in gastrointestinal cells which can recognize and efflux the therapeutic agents are other obstacles. Co-administration of p-gp inhibitors with the active therapeutic agent can decrease the efflux of genetic material, but the problem is that the inhibitors themselves exhibit toxicity in vivo (30). Nuclease-mediated degradation is another challenge, which can limit efficacy of genetic medicines. Nucleases are released from pancreas into the small intestines. There is no general agreement on bioavailability of orally used genetic material, though it has been suggested that oral administration of naked nucleic acids results in bioavailability values from 25 to less than 1 percent (2, 3). In vitro CaCo2 cell and rat inverted sac models have shown that both trancytosis and paracellular routes may be involved in the transepithelial transport of genetic material in the GI tract (2, 3). 

Liposomes are able to protect nucleic acids from degradation in the gastrointestinal tract and prolong exposure to mucosal membrane that can lead to higher concentration at the absorption site (31). Liposomes also face different stability problems in gastrointestinal tract, such as low pH values in the stomach, enzymatic action of lipases and detergent action of bile salts. Even if they survive this harsh environment, their absorption through GIT epithelium will be another obstacle. The most viable mechanism of liposome is adsorptive endocytosis and the retentive property of the particles at the absorption site (29). Liposomes may be taken up by membranous cells on the surface of GI lumen and be transported to lymphocytes in the form of vesicles. The lymphatic absorption bypasses presystemic metabolism in the liver and provides a chance to target the lymphatic system (32). In a comparison study, for vaccination via M cells, it has been shown that chitosan-coated Polyplex loaded liposomes demonstrated high potential of DNA delivery to the distal intestine in consequence of the extended stability of surface charge of the liposome containing plasmid pRc/CMV-HBs (green fluorescence protein) which were substantially important for oral DNA vaccine delivery (29).

*Ocular delivery*

Studies were also carried out to examine the local delivery of therapeutic molecules encapsulated within liposomes as a potential treatment for ocular inflammation (33). The target of gene therapy often is the posterior region of the eye. While gene delivery through the sclera is interesting, transcorneal permeation of free genetic material has not been very successful, because hydrophilicity, high molecular weight of genetic materials and their anionic nature restrict passing through the epithelial pores of the cornea. It has been shown that administration of liposomal oligonucleotides results in low concentrations in ocular tissues as compared with free delivery owing to short residence time of liposomes on the eye surface, which is not adequate to allow the release of genetic material through pores of the corneal epithelium (34, 35). 

As for other administration modalities, naked genetic material can be degraded and requires protection in the intraocular tissue. One solution can be intravitreal injection of nanocarriers that are able to protect genetic material and facilitate increased cellular delivery as a result. To avoid toxicity and difficulty related to repeated administration of intravitreal injections, the delivery system should stay in the site of application for a sufficient period to enable delivery of therapeutic doses (36). Intravitreal injection of a model phosphodiester oligonucleotide delivered via pegylated liposomes in rabbit eyes in order to decrease the degradation rate of native oligonucleotide, and increase oligonucleotide half-life within the vitreous humor, resulted in sustained release of the ODN into the vitreous and the retinachoroid compared with the solution. Also distribution to non-target tissues such as sclera and lens was reduced (37). These studies show that for good therapeutic effects, liposomes should be injected to intraocular tissues and the release from liposome should be optimized.

In a successful example of ocular gene delivery, plasmid DNA administered by pegylated liposomes has been shown to efficiently transfect retinal pigment epithelium (38). Although intravitreal liposomes are still highly investigational, progress is being made toward use in the clinic (38). There are also some reports of in vivo transfection of retinal cells with liposomal vectors. Intravitreal and subretinal injections of HVJ liposomes (hemagglutinating virus of Japan) containing LacZ gene in rat led to β-galactosidase activity in neurons and glial cells. No inflammation or toxic effects secondary to this application were detected on histologic examination. Studies indicated that Intravitreal injection of non-viral nucleic acid nanoparticles has been considered as a safe and promising approach in ocular gene transfer. Intravitreal injection of non-viral nucleic acid nanoparticles should be stable and mobile in the vitreous (39-42). 

It has also been shown that a combination of ultrasonic treatment (1.2 W/cm^2^, 20 s, duty cycle 50%) with liposomes composed of polyethylene-glycol, distearoyl phosphatidyl ethanolamine (DSPC), and perfluoropropane gas, provided a 60% increase in expression of green fluorescent protein (GFP) plasmid DNA in rat eyes when ultrasound was applied when compared to Lipofectamine 2000 transfection (43). Liu et al. have successfully demonstrated that 132 nm pegylated liposome-protamine-hyaluronic acid nanocarriers loaded with siRNA targeted against VEGFR1 not only enhance VEGFR1 knockdown, but also accelerate intracellular delivery to human RPE cells over free siRNA in vitro. After intravitreal administration, these nanocarriers were also able to significantly reduce the area of choroidal neovascularization (CNV) in a laser-induced murine CNV model with minimal toxicity, suggesting their suitability for clinical applications (44).

*Nasal gene delivery*

Nasal delivery is a useful delivery route in vaccination. The nose is the first point of contact with inhaled pathogens, rich in lymphoid tissue and has a relatively large surface area through which uptake of antigenic material can take place. This route is easy to access and eliminates the use of needles. Both systemic and mucosal immunity can be achieved following nasal vaccination in animal and human (44). Nasal administration is a noninvasive route for gene delivery (45). Beside systemic or CNS delivery, this route of administration can be employed in treating disorders of respiratory tract like chronic obstructive pulmonary disease (COPD), cystic fibrosis, asthma and viral infections of the lung (46). Individual and specific attention and requisites must be considered for each of these targets (47).

There are some barriers for liposomal gene delivery in nasal route. The nasal epithelium (mainly the olfactory epithelium) is able to metabolize naked nucleic acid constructs. Genetic material for systemic delivery after intranasal administration passes to the circulation via nasal epithelium, which is mainly composed of ciliated columnar cells, covered with a mucus layer. The mucus covering the epithelium retains particles. The cilia beat, and microvilli and turbinate of the nasal route causes trapped particles back to the pharynx area for subsequent ingestion (48).

Liposome must first adhere to the nasal mucosal surface and then, pass through the mucus, maintain the stability of the nucleic acid and release it slowly at the target site (48). 

There are documented cases, which confirm the success of liposomal gene delivery via the nasal route. For instance, it has been shown that liposomal DNA has the potential of effective treatment of cystic fibrosis (49, 50). In addition, gene expression in transfected cells showed that the liposomal formulations are suitable for mucosal immunization (51, 52). In one study, in vivo liposomal gene transfer via nasal administration showed that it could be an efficacious delivery route for nucleic acid constructs into the bloodstream. Nasal administration of cationic liposomes containing the insulin gene led to higher levels of insulin secretion in type one diabetic mice (53, 54).

*Respiratory gene therapy*

An ideal carrier for lung therapy needs to be stable against shear forces during nebulization, diffuse in the mucus layer of conducting airways and surfactant-containing liquid layer in the alveoli, overcome binding of macromolecules to the surface of the nucleic acid-containing carriers (it can lead to aggregation and therefore reduction of nucleic acid transfer capacity) and escape from macrophages, mucociliary transport or coughing, and finally permeate the barrier or release their contents for local or systemic delivery (1).

Many investigations have been performed on liposomal gene delivery to lung cell or lung epithelial barrier. The influence of surfactant lipids on the particle characteristics upon nucleic acid delivery to the lung may alter their transfection efficiency. Both synthetic and naturally derived surfactant preparations results in a dose-dependent transfection inhibition of cationic liposome (55-57). This inhibitory effect seems to be from disintegration of the liposome and subsequently nuclease-mediated degradation of genetic material (1, 58). 

As far as clinical application is concerned, it has been shown that for infectious diseases like viral infection (influenza), the results were promising and viral titers were reduced significantly (10). However, for other pathologies, successful reports of genetic transfer are yet to come. For example in an in vivo gene delivery to lungs of mice, Genzyme lipid (GL67; a new liposome formulation) was used. Related siRNAs, which were targeted to β-galactosidase, reduced this reporter gene mRNA levels in the airway epithelium of K18-LacZ mice by 30% while most of liposomes accumulated in alveolar macrophages (59). The UK Cystic Fibrosis Gene Therapy Consortium demonstrated proof-of-concept of gene transfer to the lower airways via repeatedly administration of non-viral gene transfer agent (GTAs). This approach was moving forward into a multidose clinical trial (60).

*Transdermal delivery*

As a primary and non-specific barrier to chemicals and infections, the skin is very effective when intact. It also acts as a pathway for dermal and transdermal delivery of drugs. Within this barrier, the stratum corneum (SC) is the main barrier to permeation of drug molecules and nanoparticles (61). Penetration of relatively large molecular weight and charged molecules (like genetic material) across intact stratum corneum is known to be very limited (62). In spite of this, liposomes, especially those that are deformable can accumulate in this barrier and pass through skin. Therefore, these nanoparticles are used for dermal and transdermal drug delivery and have been shown to improve drug accumulation in skin and its compartments (for example hair follicles) and to implement systemic delivery. The skin as an administration route for therapeutic genes (e.g. through the skin gene vaccination) can be a valuable alternative for systemic delivery (63), although it is currently limited due to low permeability of the SC, as above mentioned (64). Topical gene delivery is a promising technique, especially for the treatment of local skin disorders including skin carcinoma, melanoma, psoriasis and viral diseases (e.g. herpes simplex) (65). 

As well as conventional phospholipid liposomes (CLs), novel generation of liposomes (e.g. ethosomes, elastic deformable vesicles, niosomes and transfersomes) have been developed for enhanced gene delivery to transdermal targets (66, 67). Topical delivery of liposomes containing plasmid DNA encoding the β-galactosidase gene to mouse skin and to human skin xenografts was efficient in vivo. In this survey transfection efficacy of nine commercially available cationic liposome preparations in freshly isolated human hair follicles, placed in explant culture with a reporter plasmid (pSV-β-galactosidase; pSV-β-gal), were investigated. The pFx-1-DNA mixture liposomal formulations transfected 73 ± 12% of hair follicles. Liposome composition was found to have substantial effect on transfection efficacy. A lipoplex has been introduced for delivery of the gene encoding the green fluorescent protein to HeLa cells that has resulted in nuclear internalization and transfection (68, 69). In a comparative study for delivery of luciferase and β-galactosidase plasmids to rat skin, it has been shown that nonionic liposome and cationic liposome could be significantly more efficient than a liquid carrier (polypropylene glycol:ethanol:water mixture). 

Finally, in transdermal delivery, the liposomal carrier for gene delivery to the skin should guarantee non-toxicity, long-term stability, and permeation efficacy for drugs, also it is importance to develop the vehicles of well-defined intrinsic properties, such as molecular weights, HLB, chemical composition, topology, specific ligand conjugation and to investigate the effects of the properties on drug permeation behavior (70, 71).

*Systemic Barriers*

After systemic administration of liposomes containing genetic material, liposomes should stay intact in the blood, have little or no interaction with serum proteins, erythrocytes and other cellular components and be able to reach the target tissue (72). Genetic material has a short half-life in blood circulation because of rapid degradation by nucleases (73, 74). Substantial chemical modification of antisense molecules has overcome this obstacle. PS (ﬁrst-generation phosphorothioate) oligonucleotides, MOE–PS–MOE (2'-O-methoxyethyl-phosphorothioate-2'-O-methoxyethyl) gapmers, LNA (locked nucleic acid) oligonucleotides and LNA–DNA–LNA gapmers are stable in serum for extended periods, whereas OMe–PS–OMe (2'-O-methyl- phosphorothioate-2'-O-methyl) gapmers show moderate serum stability in vivo. PMO (phosphorodiamidate morpholino) oligomers show good serum stability in rats after intravenous injection. The extent of modiﬁcation is an important factor. Fully-modiﬁed PS being more stable than partially-modiﬁed PO/PS sequences but the more modification of antisense nucleotides the more reduction in sequence specificity for the target mRNA is observed (75, 76).

Cationic liposomes are able to partially or fully protect associated oligonucleotides from degradation by serum nucleases and via selective delivery to target sites (77). It has been shown that intravenous application of liposomes have more significant effect than naked DNA as these carriers prevent degradation of genes and promote cellular uptake (78, 79).

In the case of the more commonly used cationic liposome-mediated nucleic acid delivery, the positive charge of the resulting complex (lipoplex), besides its benefits, also enhances non-specific electrostatic interactions of liposomes with serum components and molecules which result in decrease of subsequent transgene expression in vivo (24). The most important obstacle for liposomal nucleic acid delivery is the serum, a complex ﬂuid containing lipoproteins, enzymes such as lipases and nucleases that can degrade liposomes and the genetic payload and therefore interfere with transfection efficiency (80-82).

Liposomes with neutral or anionic surfaces show enhanced stability in serum and increased circulation time but low loading of genetic material and reduced uptake by target cells, making them inferior to cationic liposomes. 

Other surface components and properties of liposomes are also important. In vitro experiments have shown that the glyco-coated liposomes are efficiently taken up by cells expressing carbohydrate-binding receptors selectively. (83-86). Biodegradable agents such as polyhydroxyethyl L-asparagine/L-glutamine and polyethylene glycol (PEG) attached by a hydrolysable bond such as an ester to the liposome surface have the advantage of prolonged circulation in serum and diminish binding to the cell surface or deleterious opsonisation, and binding and internalization to target tissue and cells (75, 87). Incorporation of PEG into the liposome diverts its accumulation in the lung to distal solid tumors (88). It has been shown that for in vivo gene delivery via upstream intra-arterially administration, tumor uptake can be enhanced by docking of liposomes on to microspheres (25).

To reach the interstitial spaces of tumors, liposomes must pass the 50–100 nm thick glycocalyx shield on the luminal side of endothelial vessel first (89). Relatively high interstitial ﬂuid pressures and the organization of the interstitial environment are hurdles that keep liposomes from accumulating in target cells at high concentrations. One other obstacle in blood is clearance from the blood by the kidney and reticuloendothelial system (RES: lungs, liver and spleen) and extravasation in organs other than those constituting the RES (75). Attachment of some hydrophilic components such as PEGs to the liposome can reduce uptake by RES.

Even after upstream intra-arterial administration for genetic drugs, limited targeting and selectivity for cancer has been achieved (90). Manipulation of liposomal structure and composition (example ligand-receptor binding) has been known to promote specific delivery or targeting. Without targeting moiety, deposition in capillary beds of the lung and subsequent release into the plasma and clearance by spleen and liver due to its size and high charge is likely a big obstacle in many cases (91). Targeting ligands can be added either by directly coupling the ligand to the phospholipids or distal end of the PEG-lipid; more accessible for interaction with the receptors (80).

There are many examples for targeting. In one study, plasmid DNA encoding glial-derived neurotropic factor (GDNF) was encapsulated into Trojan horse liposomes (THLs) with a monoclonal antibody (MAb) to the rat transferrin receptor (TfR) that has shown good therapeutic efﬁcacy in brain (63). Other targeting ligands including galactose (92) or asialorosomucoid (93), mannose (94) folate (95) or transferrin (96) ligands for uptake by cells expressing the folate or transferrin receptor and cytoskeleton speciﬁc ligands for targeting injured cells have been studied (88). However, studies are necessary to assess in vivo, whether such targeting or selective delivery of nucleic acids actually occurs.

*Cellular* *barriers*

**Figure 2:**
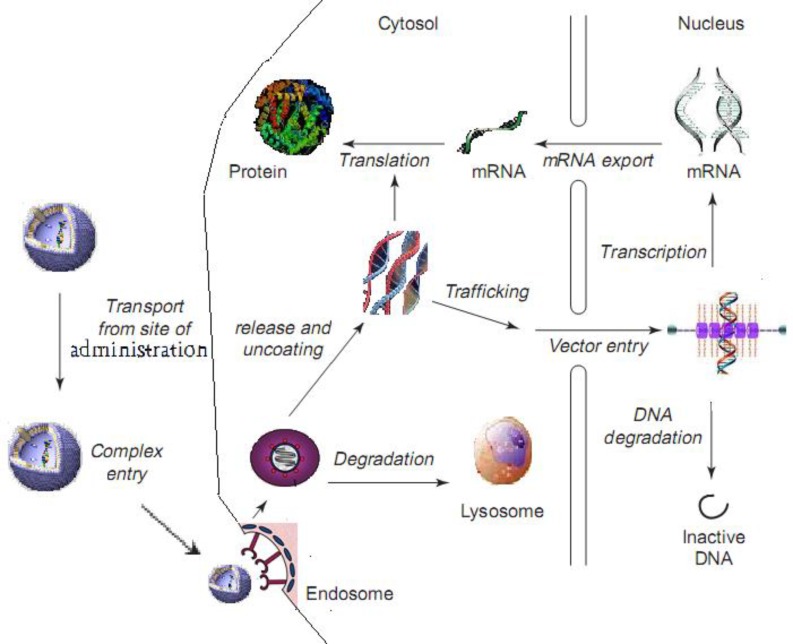
Possible pathways and barriers of liposomal gene delivery at cellular and subcellular levels

When liposomes reach a target cell, it has to overcome certain barriers for successful transfection. These barriers include (a) binding of the liposome to the cell surface, (b) entry of the liposome into the cells by endocytosis or direct traversing of the plasma membrane (e.g. via membrane fusion), (c) escape of the liposome from the endosome, (d) dissociation of the liposome to release nucleic acid payload, (e) transport through the cytosol and (f) entry into the nucleus (Figure 2), as discussed below.

*The* *initial binding and entrance*

Cationic liposomes adhere to cells via non-specific electrostatic interactions with the cell surface. Some viruses exploit similar mode of interaction to enter target cells (97). Endocytosis and/or direct fusions with cell are two main approaches for liposomes to access the cell interior that can be impressed by preparation method (98). Adequate cationic charge on the surface of formulated liposomes is essential for optimal delivery into the cell (99). Generally, unshielded highly cationic liposomes enter cells through nonspecific endocytosis mechanisms like as macropinocytosis and phagocytosis. Simple charge interactions with cellular networks of polyanions also may have role in internalization. Shielding liposomes can be used to reduce uptake by non-target cells. Furthermore, ligands or antibodies can be added to the surface of liposomes to promote correct cell-specific attachment and receptor-mediated uptake (75). Physical method like gene gun, radiation (100), electroporation and sonophoresis (101, 102) can enhance liposomal gene delivery via higher entrance to target cells.

Different ligands have been used for binding and facilitating endocytosis (103). In a survey, binding and internalization of siRNA-loaded immunoliposomes (containing anti-CD33 single-chain Fv fragment) to leukemic cell lines were evaluated by ﬂuorescence microscopy using labeled siRNA. A highly antigen-speciﬁc uptake into CD33-positive SKNO-1 and Kasumi-1 cells was observed while unconjugated liposomes showed a very weak binding to the same cells (104). Another survey reported that modified C16Y peptide on nanoliposomes, may be a feasible approach to target endothelial and cancer cells via the integrin receptor (105). Several classes of targeting ligands, including proteins, vitamins, carbohydrates, hormones and monoclonal antibodies have been used for targeted liposomal gene delivery. Some ligands also may reduce surface charge of liposomes and reduce non-specific uptake. Two different ligands or more may be attached to a liposome to create heterovalent ligand-attached vector constructs capable of binding to multiple receptors. Usually one acts to target a surface antigen and the other ligand targets a highly specific internalizable receptor (75). In a study examining functionalized nanoparticles with two angiogenesis-specific targeting ligands, an α_v_β_3_ integrin-specific and a galectin-1-specific peptide, the uptake of nanoparticles was increased when compared to nanoparticles using single ligand targeting (106). One area that deserves more survey is mechanistic studies to find out exactly how liposomes are internalized and genetic material released to the action site (107, 108). In contrast, mechanisms of how constructs down regulate or overexpress a gene are well studied.

*Escape from the* *endosome/lysosome compartment*

After internalization of the liposome, the most challenging step in gene delivery is release of the genetic construct from the endosomes to the cytoplasm. The liposomal contents should be able to escape from the endosome and be free of liposome in adequate quantities. The endosomal escape mechanism most often is based on the disruption of endosomal membrane. A number of strategies have been suggested to enhance the release of genes from endosomes, that are discussed below.

One of the strategies for endosomal escape is use of endosomotropic agents like chloroquine that accumulate in and buffer the pH of endosomes, which leads to higher release of vectors (109). However, effective concentrations of chloroquine and similar lysosomotropic reagents are toxic to humans. One other method is the application of pH-sensitive fusogenic proteins, which usually are obtained from viruses (for example, the hemaglutinin subunit HA-2 from the influenza virus). This agent changes in the acidic endosome, thereby interacting with and perturbing the endosome (110, 111). Also the translocation domains of the diphtheria and anthrax toxins or amphipathic sequences such as GALA (a peptide with a glutamic acid-alanine-leucine-alanine repeat) can deliver genetic material to the cytoplasm and act at the low pH of the early endosome via membrane fusion and permeabilization (112, 113). Another technique consists of the use of anionic pH-sensitive liposomes [oleic acid/DOPE or CHEMS (cholesteryl hemisuccinate)/DOPE] which are known to undergo phase transition and lipid fusion when the pH is lowered and the acidic head-group is neutralized in endosomes, thereby facilitating gene delivery to cytoplasm (114, 115). This advantageous property is attributed to the neutrally charged DOPE as co-helper lipid (116). Proton sponge compounds such as PEI destabilizes the endosomal compartment and allows release of the nucleic acid into the cytoplasm (117, 118). 

**Figure 3:**
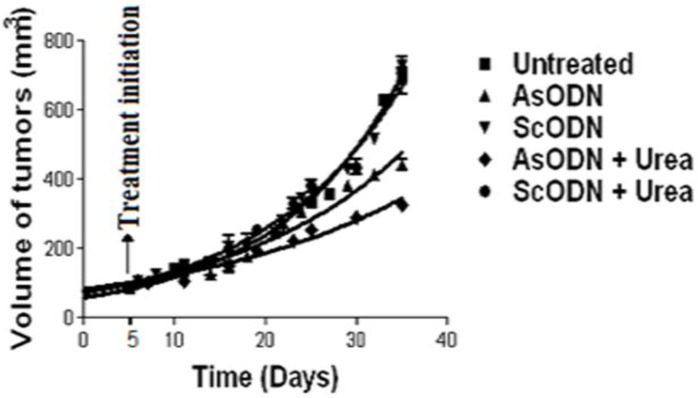
Tumor growth profile of different antisense (As) or scrambled (Sc) oligodeoxynucleotides (ODN) liposomal formulations in the presence or absence of urea in nude mice in comparison to untreated control animal. Data are mean ± standard error (n = 3). (From Ref. 119, with permission

Destabilization of endosomal lipid bilayers by chemical penetration enhancer, has been investigated in our laboratories and seem to be an efficient solution to overcome release obstacle in liposomal gene delivery. In-vivo evaluation of this theory in nude mice lead to significant decrease of xenograft tumor growth (as shown in Figure 3) when liposomal gene delivery combined with urea solution or cineole as a chemical enhancer (119, 120).

*Trafficking in the cytoplasm*

Genetic material may work in the cytoplasm (example interaction with mRNA) or might be required to enter the nucleus for action. Owing to poor diffusion, reaching the nucleus for large genetic molecules in the highly arranged and complex medium of the cytoplasm is hard. This diffusion is size-dependent (121). As well as macromolecular crowding, dense sterical hindrances by the cytoskeleton reduce diffusion of the usually bulky genetic material. In the context of normal intracellular trafficking, endogenous proteins, organelles, and vesicles are transported along the cytoskeletal network, akin to a rail system. Liposomes may be trafficked by microtubes in the cytoplasm (75). There are different microtube pathways in a cell and if a liposome can become part of the bidirectional microtubes leading to and from the nucleus, perhaps nuclear delivery could be enhanced. For instance, attachment of a suitable ligand (example, dynein-association sequences) can enhance cytoplasmic transport to the perinuclear region (75). Our studies have shown that some material (such as urea) might help cytoplasmic transport of liposomal oligonucleotides and help the material to get closer to the nucleus (119).

*Transport to the nucleus*

In the case of plasmid DNA, the gene, before being expressed into the therapeutic protein, has to reach the cell nucleus to gain access to the transcription machinery. To enter the nucleus, molecules must pass through nuclear pore complexes. DNA fragments, which are larger than 300 base pair cannot passively diffuse into the nucleus since they are larger than the upper molecular weight cut-off of nucleus membrane for passive entry (122). 

During cell division, the nucleus membrane is temporarily non-continuous and therefore can be breached (122). In addition, proteins that normally localize to the nucleus possess a specific targeting signal called the nuclear localization sequence (NLS) (123). Numerous studies have attempted to enhance nuclear import of liposome or other nonviral vectors by addition of an NLS to the non-viral vector. The minimal NLS (PKKKRKV132) of the simian virus SV40 large tumor antigen (T-ag) has been used frequently in this regard. The minimal T-ag NLS has been shown to cause higher expression of the transgene when conjugated or form complex directly with genetic cargo. It has been shown that using T-ag NLS peptide (T-ag residues *126-135*) conjugated to the end of a linear DNA fragment condensed in a cationic liposome can induce nuclear uptake (124-125). NLSs like GAL4 (126, 127) and opT-NLS (128) also were able to increase expression of the transgene.

## Conclusion

Liposomes are promising nanocarriers in gene therapy. The success of these systems depends on the liposome properties, administration route and the barriers that they face to reach their target inside the cells. These hindrances include, but not limited to, stability in the site of administration, permeation of particles through epithelial barriers, stability in the bloodstream, low target cell specificity, especially when applied through routes that are far from the target, escape from the RES, uptake by the cells and access to the appropriate site within the cytosol or nucleus. For a successful liposomal gene delivery, these barriers and the nature of liposome-cargo-barrier interaction should be well investigated and understood. As discussed in this review, some of these barriers have been overcome in some way; however, we should note that the problem is yet to be solved completely.
